# Employer-Sponsored Fertility and Family-Building Benefits Among 928 U.S. Employers

**DOI:** 10.7759/cureus.116546

**Published:** 2026-09-19

**Authors:** Kulsoom Farooqi Baloch, Veera Saghar, Rashmi Gulati

**Affiliations:** 1 Medicine, Ziauddin University, Karachi, PAK; 2 Public Health, Hofstra University, Hempstead, USA; 3 Research – Reproductive Health and Family-Building Benefits, Isra University, Karachi, PAK; 4 Internal Medicine, Mount Sinai Hospital, New York, USA

**Keywords:** assisted reproduction, employer health benefits, employer-sponsored benefits, family-building benefits, fertility benefits, fertility coverage, gestational surrogacy, health policy, in vitro fertilization, reproductive health

## Abstract

Background

Employer-sponsored fertility and family-building benefits are increasingly used to finance in vitro fertilization, fertility preservation, adoption, donor services, and, in some plans, surrogacy-related expenses. However, employer-level benefit design and financial limits are not consistently reported in publicly available sources. This study aimed to characterize publicly reported U.S. employer fertility and family-building benefits in 2026, focusing on benefit amounts, benefit design, administrators, and source quality.

Methodology

We conducted a cross-sectional descriptive analysis of publicly available employer-level benefit information compiled through August 12, 2026. Records were drawn from national employer-benefit surveys, employer-benefit directories, the Men Having Babies Workplace Benefits Database, published employer-benefit compilations, vendor information, and employer or program sources where available. Employer names were standardized and exact-name duplicates were removed. The analytic master dataset contained 928 unique employers. Exact-dollar analyses were restricted to the 67 (7.2%) employers with a standardized numerical family-building or surrogacy-related amount matched to an employer in the master dataset; categorical estimates were retained separately and were not converted to exact values. No missing values were imputed.

Results

The analytic master dataset included 928 unique employers, comprising a 664-employer base cohort and 264 employers added during a later directory expansion. The exact-dollar analytic subset consisted of 67 employers (7.2% of 928) with a standardized exact family-building or surrogacy-related amount. Among these 67 employers, the median reported amount was $25,000 (interquartile range = $20,000-$40,000), with a range of $2,500-$150,000; 54 of 67 (80.6%) had reported amounts of at least $20,000, 14 of 67 (20.9%) had amounts of at least $50,000, and six of 67 (9.0%) had amounts of at least $75,000. A named benefit administrator or program was publicly identified for 29 of 928 (3.1%) employers; Carrot, Maven, Kindbody, and Progyny were the most frequently named specialty vendors. Evidence quality was heterogeneous: most exact dollar amounts were supported by secondary or directory sources rather than current employer plan documents.

Conclusions

Publicly reported employer fertility and family-building benefits vary substantially in amount and design. Among employers with exact numerical amounts available, the findings underscore that offering a family-building benefit does not necessarily indicate comprehensive financial protection because benefit magnitude and covered services vary. Future research using primary plan documents and utilization data should examine how benefit design affects access, out-of-pocket spending, and treatment decisions.

## Introduction

Access to assisted reproductive technologies in the United States is strongly influenced by benefit design and out-of-pocket cost. Employer-sponsored coverage is especially important because fertility services may be excluded from standard medical coverage, subject to lifetime or cycle limits, or delivered through a separate specialty benefit. National employer surveys demonstrate that in vitro fertilization (IVF) coverage has expanded during the past several years. Mercer reported that 47% of employers with 500 or more employees covered IVF in their largest medical plan in 2024, compared with 22% in 2019 [1]. The International Foundation of Employee Benefit Plans (IFEBP) reported that 42% of U.S. organizations offered fertility benefits in 2024 and 32% offered IVF [2]. KFF, a nonprofit health policy research, polling, and news organization, separately reported IVF coverage among 27% of firms with 200 or more workers that offered health benefits in 2024 [3]. Differences across surveys reflect distinct sampling frames and definitions and should not be interpreted as directly comparable prevalence estimates.

Employer family-building programs may extend beyond medically indicated infertility treatment to fertility preservation, donor services, adoption, and surrogacy-related expenses. However, employer-level plan details are difficult to characterize systematically. Benefit amounts are frequently described in recruiting materials, benefits directories, vendor pages, trade publications, consumer resources, or media reports rather than in publicly available Summary Plan Descriptions. Published values may therefore be outdated, may combine multiple family-building pathways, or may conflict across sources.

The regulatory environment is also evolving. State infertility coverage requirements vary substantially and generally do not reach self-funded employer plans in the same manner as state-regulated insurance [4,5]. In May 2026, the U.S. Departments of the Treasury, Labor, and Health and Human Services proposed a new category of excepted fertility benefits with a proposed maximum lifetime benefit of $120,000, subject to defined scope and plan-design requirements [5]. This proposed ceiling provides policy context for the exact-dollar distribution examined in this study, although the proposed federal benefit and the broader family-building amounts in our dataset are not directly equivalent.

This study aimed to describe a large convenience dataset of publicly reported U.S. employer fertility and family-building benefits as of August 2026. The analysis focused on the completeness and distribution of reported benefit amounts, benefit architecture and administration, and the quality and limitations of publicly available employer-level evidence. It was not designed to estimate the national prevalence of fertility or surrogacy benefits among U.S. employers. By characterizing the magnitude, structure, and transparency of disclosed employer-level benefits, this study complements national employer surveys that primarily estimate whether coverage is offered.

## Materials and methods

Study design and data freeze

We conducted a cross-sectional descriptive analysis of publicly available, employer-level secondary data. The source compilation was maintained as an evolving working dataset, and a single prospective study-start date was not recorded; for reproducibility, the analytic master file was frozen on August 12, 2026. The master contained 928 unique employer names after standardization and exact-name de-duplication. The dataset consisted of an earlier 664-employer working cohort and 264 additional employers incorporated during a later expansion using the Men Having Babies Workplace Benefits Database [6]. Records previously present in the base cohort could also contain Men Having Babies as a corroborating source; the 264 figure refers only to newly added employer records.

Data sources

Employer records were compiled from several source types: national employer-benefit surveys and benchmarking reports [1-3]; a national employment-perks directory [7]; the Men Having Babies Workplace Benefits Database [6]; published employer-benefit compilations and consumer or industry resources [8-11]; specialty-benefit vendor information; and official employer or program information when publicly available. The federal regulatory analysis was based on the May 13, 2026 Federal Register proposed rule [5]. Each employer record retained the source URL or source description used in the working dataset whenever available.

Variables and definitions

Variables included employer name; IVF or fertility benefit description; IVF dollar or cycle limits; fertility-medication limits; cryopreservation; donor services; surrogacy-specific limits; adoption limits; combined or other family-building limits; named benefit administrator or program; eligibility notes; verification status; source; and source year or date checked. The workbook also contained operational prioritization fields used outside the research analysis; those fields were excluded from the publication dataset.

For quantitative analysis of family-building or surrogacy-related amounts, we used only standardized exact numerical values that could be matched to an employer in the 928-employer master dataset. Overall, 67 of 928 (7.2%) employers met this criterion; therefore, 861 of 928 (92.8%) lacked a standardized exact numerical value usable for the exact-dollar analysis. Publicly reported categorical estimates, including broad lifetime-benefit bands from directory sources, were preserved as categorical information and were not transformed into exact dollar values. Values described only as “included,” “shared,” “not publicly stated,” or otherwise non-numeric were treated as missing for exact-dollar analyses. No imputation was performed.

Evidence class was derived from the verification language already present in the working dataset rather than from a prospectively validated source quality scale. “Official/primary” indicated information attributed to an employer or official employer source; “primary program confirmation with a secondary dollar amount” indicated primary confirmation of the program with the dollar value obtained from a secondary source; “multiple or conflicting secondary sources” indicated more than one secondary report with conflicting or alternative values; “secondary” indicated information attributed to a secondary published source; “directory/secondary” indicated information derived from an employer-benefit directory or similar compilation together with secondary source documentation; and “unverified/public-directory” indicated directory-reported information without documented primary verification in the working dataset. These categories describe source provenance and recorded verification status and should not be interpreted as a validated hierarchy of source quality. Employer record data curation and standardization were performed by K.B., with validation and investigation of the compiled data performed by V.S.; R.G. provided supervisory and clinical review. Records with explicitly conflicting reported benefit limits or alternative source values were retained and flagged as conflicting evidence rather than resolved by selecting the highest or most favorable reported amount.

Statistical analysis

Analyses were descriptive. We report counts and percentages for data availability and selected benefit characteristics. Exact reported dollar amounts are summarized using the median, interquartile range (IQR), minimum, maximum, and prespecified dollar bands. Descriptive statistical analyses were performed using SPSS Statistics (IBM Corp., Armonk, NY, USA). Because the employer dataset is a convenience sample assembled from public directories and benefit compilations, no inferential estimates of national employer prevalence were calculated from the 928-employer denominator. National prevalence estimates are reported only from independent employer surveys [1-3].

Ethical considerations

The study used publicly available employer-level information and did not involve identifiable private information about human participants or collection of human subject data.

## Results

Dataset characteristics

The final master dataset contained 928 unique employers. The publication dataset included all 928 employer records and preserved missing or nonpublic plan information rather than treating the absence of a reported field as the absence of a benefit. The 928 records comprised 664 employers in the base working cohort and 264 employers added during the later directory expansion.

Detailed plan information was sparse. A standardized exact family-building or surrogacy-related dollar amount could be matched to 67 employers (7.2% of the dataset). This proportion reflects public data availability, not the prevalence of dollar-denominated benefits. The remaining records included categorical benefit estimates, benefit descriptions without a public dollar limit, or incomplete information. Descriptive characteristics of the dataset and the exact-value subset are summarized in Table 1.

**Table 1:** Descriptive characteristics of the 928-employer dataset and the subset with standardized exact dollar amounts. Dollar statistics are calculated only among the 67 employers with matched exact numerical values; they should not be interpreted as national employer prevalence estimates.

Measure	Result
Unique employers in the final master dataset	928
Base working cohort	664
Later Men Having Babies (MHB)-added employers	264
Employers with standardized exact family-building/surrogacy amount	67 (7.2%)
Minimum exact reported amount	$2,500
25th percentile	$20,000
Median	$25,000
75th percentile	$40,000
Maximum exact reported amount	$150,000
Exact amount ≥$20,000	54 (80.6% of 67)
Exact amount ≥$50,000	14 (20.9% of 67)
Exact amount ≥$75,000	6 (9.0% of 67)

Distribution of exact reported family-building amounts

Among the 67 employers with a standardized exact numerical family-building or surrogacy-related amount, the median was $25,000 (IQR = $20,000-$40,000), and the range was $2,500-$150,000. Three of 67 (4.5%) had reported amounts below $10,000, 10 of 67 (14.9%) were in the $10,000-$19,999 range, 40 of 67 (59.7%) were in the $20,000-$49,999 range, eight of 67 (11.9%) were in the $50,000-$74,999 range, and six of 67 (9.0%) had reported amounts of at least $75,000. Overall, 54 of 67 (80.6%) had amounts of at least $20,000. The distribution of standardized exact reported amounts is presented in Table 2.

**Table 2:** Distribution of standardized exact family-building or surrogacy-related benefit amounts among employers with a numerical value available in the working dataset.

Reported exact amount	Employers, n (%)
<$10,000	3 (4.5%)
$10,000–$19,999	10 (14.9%)
$20,000–$49,999	40 (59.7%)
$50,000–$75,000	8 (11.9%)
≥$75,000	6 (9.0%)
Total with exact amount	67 (100%)

Benefit administration

A named benefit administrator or program was publicly identified for 29 of 928 (3.1%) employer records. Among these 29 records, Carrot was named in 14, Maven in five, Kindbody in four, and Progyny in three; three records named another program or administrator. Because administrator information was absent or described as not publicly stated for most employers, these counts characterize only the subset with an identified administrator and do not establish market share.

Source quality and verification

The employer-level evidence base was dominated by secondary and directory sources. Across all 928 records, the working verification language classified 701 as unverified/public-directory information, 107 as directory/secondary, 97 as secondary, 14 as multiple or conflicting secondary sources, three as primary/official employer sources, one as a primary program confirmation with a secondary dollar amount, and five as other or mixed classifications. These categories reflect the dataset’s documented verification status rather than an independent audit conducted for this investigation.

Among employers with exact numerical amounts, secondary reporting remained the predominant evidence source. Conflicting values were retained as a data quality concern rather than reconciled by selecting the most favorable or largest reported amount. For example, where the working notes identified alternative benefit limits across sources, the record was flagged for plan-level verification.

## Discussion

This cross-sectional analysis demonstrates both the scale and the limitations of publicly available employer-level information on fertility and family-building benefits. The final working dataset contained 928 unique employers, but detailed numerical plan information was available for only a small subset. Among 67 employers with a standardized exact numerical family-building or surrogacy-related amount, the median reported amount was $25,000, and values varied widely from $2,500 to $150,000. The concentration of exact values between $20,000 and $49,999 suggests that publicly reported employer assistance often represents a meaningful but bounded financial contribution rather than comprehensive coverage of every family-building pathway.

The analysis also highlights a distinction that is often obscured in consumer-facing discussions: fertility treatment, family-building wallets, adoption assistance, and surrogacy reimbursement are not interchangeable benefit categories. A combined family-building allowance may be shared across several services, whereas an IVF benefit may be delivered through the medical plan or a specialty vendor and may be limited by dollars, cycles, network rules, or clinical criteria. Public sources frequently omit these distinctions. Accordingly, counseling based solely on a headline dollar figure may overstate what an employee can actually use for a particular service.

A second major finding is the weakness of employer-level public documentation. Only 67 of 928 employers had a standardized exact amount available in the working dataset, and most exact amounts were supported by secondary sources rather than current plan documents. This finding should not be interpreted to mean that the remaining employers lack financial benefits; rather, it indicates that plan terms are often unavailable in public sources. The same problem affects administrator information: a named program or administrator was available for only 29 employers. These limitations support routine verification through current employer benefits materials, Summary Plan Descriptions, or vendor portals before financial counseling or reimbursement decisions are made.

Comparison with existing literature

Our findings are consistent with existing evidence that employer support for fertility and family-building care has expanded, while also showing that benefit design remains heterogeneous. Mercer reported that IVF coverage among employers with 500 or more employees increased from 22% in 2019 to 47% in 2024 [1]. The International Foundation of Employee Benefit Plans similarly reported that 42% of U.S. organizations offered fertility benefits in 2024 and 32% offered IVF, compared with 24% offering IVF in 2020 [2]. KFF reported that among firms with 200 or more workers offering health benefits in 2024, 37% covered fertility medications, 27% covered IVF, 12% covered cryopreservation, and 13% covered adoption services [3]. These studies demonstrate increasing employer involvement in family-building care, although differences in sampling frames, employer size, and benefit definitions prevent direct comparison of prevalence estimates.

The present study complements these national surveys by characterizing the magnitude and structure of publicly disclosed employer benefits rather than estimating national coverage prevalence. Among the 67 employers in our dataset with a standardized exact family-building or surrogacy-related amount, the median reported benefit was $25,000 (IQR = $20,000-$40,000). Mercer has separately reported that dollar-based IVF benefit designs are common among large employers that cover IVF, with a median lifetime dollar limit of $20,000 in its 2024 survey [1]. A later Mercer analysis reported a median maximum of $25,000 while continuing to emphasize substantial variation by employer size and benefit design [12]. Although these Mercer estimates and our broader family-building measure are not directly equivalent, the comparison indicates that financial caps in the range observed in our dataset are consistent with benefit limits reported in large employer surveys.

Our findings also reinforce the distinction between offering a benefit and providing comprehensive financial protection. KFF specifically notes that its survey identifies whether employers provide coverage for family-building services but does not establish what proportion of service costs is covered [3]. In our employer-level dataset, publicly reported amounts could apply to IVF, fertility preservation, donor services, adoption, surrogacy, or a shared family-building allowance. National surveys and employer-level directories therefore provide complementary information: surveys are better suited to estimating the prevalence of benefit availability, whereas employer-level data can help describe the financial magnitude, structure, and transparency of disclosed benefits.

The dataset also illustrates why national survey estimates and employer directories should serve different purposes. Mercer, IFEBP, and KFF provide probability-based or structured survey estimates within defined employer populations [1-3]. In contrast, the 928-employer dataset is a convenience sample enriched for organizations that publicly advertise or are reported to offer fertility or family-building benefits. It is therefore useful for describing benefit architecture, public reporting practices, and the distribution of disclosed amounts, but it cannot estimate the percentage of all U.S. employers offering IVF, surrogacy, or other family-building benefits.

The evolving federal policy environment may increase employer flexibility while also increasing the need for clear benefit definitions. The 2026 proposed excepted fertility benefit rule would allow qualifying standalone fertility coverage with a proposed lifetime maximum of $120,000 [5]. Importantly, the proposal concerns fertility benefits defined within the regulation; it should not be interpreted as establishing a federal surrogacy reimbursement entitlement. Future rulemaking and employer plan design will determine how broadly new benefit structures are used and which services are eligible.

Limitations

This study has several limitations. First, the employer dataset is a convenience sample assembled from public directories, benefit compilations, vendor information, and selected official sources; it is not representative of all U.S. employers. Second, public reporting was incomplete, and missing plan details were common. Third, most employer-level dollar amounts were not independently verified against current Summary Plan Descriptions, and some records contained conflicting secondary reports. Fourth, broad family-building amounts may cover different combinations of IVF, fertility preservation, donor services, adoption, or surrogacy, limiting direct comparability between employers. Fifth, source classifications were derived from verification language in the working dataset and do not constitute an independent source quality adjudication. Finally, the authors are affiliated with a surrogacy agency, creating a potential conflict of interest. To reduce this concern, the revised analysis does not use the agency’s program pricing to estimate the proportion of a surrogacy journey offset by employer benefits.

## Conclusions

Publicly reported employer fertility and family-building benefits in the United States vary substantially in structure and financial magnitude. In this 928-employer convenience dataset, exact numerical family-building or surrogacy-related amounts were publicly available for 67 employers, with a median reported amount of $25,000 and an IQR of $20,000-$40,000. By describing the magnitude, structure, and transparency of publicly disclosed employer-level benefits, these findings complement national surveys that primarily measure whether coverage is offered and help distinguish benefit availability from the degree of financial protection provided. Standardized, dated disclosure of covered services, eligibility criteria, dollar and cycle limits, tax treatment, and benefit administrators would improve employee counseling and future research. Studies using primary plan documents and utilization data are needed to determine how employer family-building benefits affect access, out-of-pocket spending, and treatment decisions.
